# Changes in ocular extorsion after horizontal muscle surgery in patients with intermittent exotropia coexisting with hypertropia and mild inferior oblique overaction

**DOI:** 10.1371/journal.pone.0297427

**Published:** 2024-02-05

**Authors:** Soo Jung Lee, Sook Hyun Yoon, Sook Young Kim, Donghun Lee

**Affiliations:** Department of Ophthalmology, Daegu Catholic University School of Medicine, Daegu, Korea; Aravind Eye Hospital, INDIA

## Abstract

**Purpose:**

To investigate changes in vertical strabismus and extorsion in patients with intermittent exotropia and mild unilateral inferior oblique muscle overaction (IOOA) who underwent horizontal muscle surgery without vertical or oblique muscle surgery.

**Methods:**

The medical records of 41 patients were retrospectively analyzed. The patients were followed up for at least 6 months after surgery. Fundus photography was performed before and after surgery, and the sum of the angles of torsion in both eyes was used to measure changes in extorsion using ImageJ software. The enrolled patients were divided into two groups according to the degree of IOOA: patients with grade 1 IOOA were placed in +1 IOOA group and those with grade 2 IOOA in +2 IOOA group. The pre- and postoperative angles of horizontal and vertical strabismus and extorsion were compared between the two groups.

**Results:**

The +1 IOOA and +2 IOOA groups included 24 and 17 patients, respectively. The angle of preoperative exotropia did not differ significantly: 25.54 ± 5.68 prism diopters (PD) and 25.65 ± 8.11 PD in the +1 IOOA and +2 IOOA groups, respectively. In the +1 IOOA and +2 IOOA groups, hypertropia was 2.67 ± 1.52 PD and 2.82 ± 1.13 PD, respectively, and extorsion angles were 7.14 ± 2.77° and 7.94 ± 2.87°, respectively. As the IOOA degree increased, the extent of hypertropia and extorsion also increased. However, there were no significant differences between the two groups. Postoperative angles of hypertropia and extorsion significantly decreased in both groups (p < 0.001) after surgery. The degree of change in hypertropia and extorsion was not significantly different between the two groups (p = 0.563 and p = 0.354, respectively).

**Conclusions:**

Hypertropia and extorsion improved significantly after horizontal muscle surgery in patients with mild unilateral IOOA and intermittent exotropia. There was no significant difference in the improvement in hypertropia or extorsion between IOOA grades I and II.

## Introduction

Patients with intermittent exotropia commonly experience concurrent mild hypertropia and inferior oblique muscle overaction (IOOA) [1]. Hypertropia occurs in 40–63% [2,3] and IOOA in 32% [4] of patients with intermittent exotropia.

If the accompanying vertical strabismus does not cause cosmetic or functional abnormalities, surgical correction of intermittent exotropia alone is commonly performed. Several studies have demonstrated that in patients with intermittent exotropia, mild vertical strabismus can only be reduced by means of horizontal muscle surgery [5–7].

Furthermore, Wilson and Parks reported that mild IOOA in patients with exotropia could only be improved with horizontal muscle surgery [4]. Cho and Kim reported similar results and stated that hypertropia and IOOA can be caused by long-term horizontal eye alignment abnormalities [1].

Although many studies have investigated the changes in vertical strabismus and IOOA after horizontal muscle surgery alone, no studies have examined the changes in extorsion, which is the main function of the inferior oblique muscle, after horizontal muscle surgery. Therefore, this study aimed to investigate the changes in vertical strabismus and extorsion when only horizontal muscle surgery was performed in patients with intermittent exotropia coexisting with hypertropia and mild inferior oblique overaction.

## Materials and methods

Patients diagnosed with intermittent exotropia and unilateral IOOA at the ophthalmology clinic of the hospital and those who underwent horizontal muscle surgery between January 2014 and June 2022 were enrolled. This retrospective study was approved by the Institutional Review Board (IRB number: CR-23-026-L) at Daegu Catholic University School of Medicine, Daegu, Korea, and all procedures adhered to the tenets of the Declaration of Helsinki. Informed consent was obtained verbally from all participants, with their guardians and residents as witnesses. Data were accessed for research purposes from March 2, 2023 to April 30, 2023.

### Inclusion and exclusion criteria

Only patients with basic intermittent exotropia, with a distance deviation within 10 diopters (D) of the near deviation, were included. Patients who underwent fundus imaging at least twice, once before and once after surgery, were included. Furthermore, both primary and secondary IOOA patients, including those with a positive Bielschowsky head-tilt test due to superior oblique palsy, were included. Patients with neurological deficits or a history of ocular trauma due to ocular surgery, including strabismus, were excluded. Patients with dissociated vertical deviation, severe amblyopia, severe refractive error over 6 D of spherical equivalent in refractive error, lateral incomitance, nystagmus, or a postoperative follow-up period of < 6 months were also excluded. Lateral incomitance with a reduction of 20% or more in lateral gaze from the primary position. Patients who underwent reoperation due to exotropia recurrence during the follow-up period or who underwent concurrent oblique muscle surgery were also excluded.

### Ophthalmologic examinations and surgical techniques

All patients underwent ophthalmological examinations, including measurement of best-corrected visual acuity, cycloplegic refractive error assessment, fundus examination, ocular motility testing, stereoacuity test, and slit-lamp examination. The angles of deviation at distance fixation (6 m) and near fixation (33 cm) were measured using the alternate prism cover test in the refractive error-corrected state. To calculate changes in eye torsion, fundus imaging was performed in all patients before and after surgery, in a non-mydriatic state, using a fundus camera (KOWA, Tokyo, Japan), with the head placed in a neutral position on a chin rest. Stereoacuity was measured using the Frisby Davis distance (FD2, Stereotest, Sheffield, UK) test. The severity of IOOA was graded according to the degree of overelevation of the eye in adduction using a +1 to +4 rating system [8].

All strabismus surgeries were performed under general anesthesia using the fornix approach. Detached muscles were sutured to the sclera. In this technique, 6–0 absorbable polyglactin 910 sutures with a spatulated needle was used and needles from the double-arm suture were passed perpendicular and then parallel to the scleral insertion line. The depth of the scleral pass is superficial; thus the needle produced a bump in the sclera. The sutures are pulled to advance the muscle, which is tied tightly in place to prevent sagging. One of three types of surgery: unilateral lateral rectus recession, bilateral lateral rectus recession, or unilateral rectus recession and medial rectus resection (R&R), was selected based on the largest angle measured.

All ophthalmological examinations were performed preoperatively and at the 1-day, 1-week, 1-month, and 6-month follow-up after surgery.

### Data evaluation

**Fig 1:**
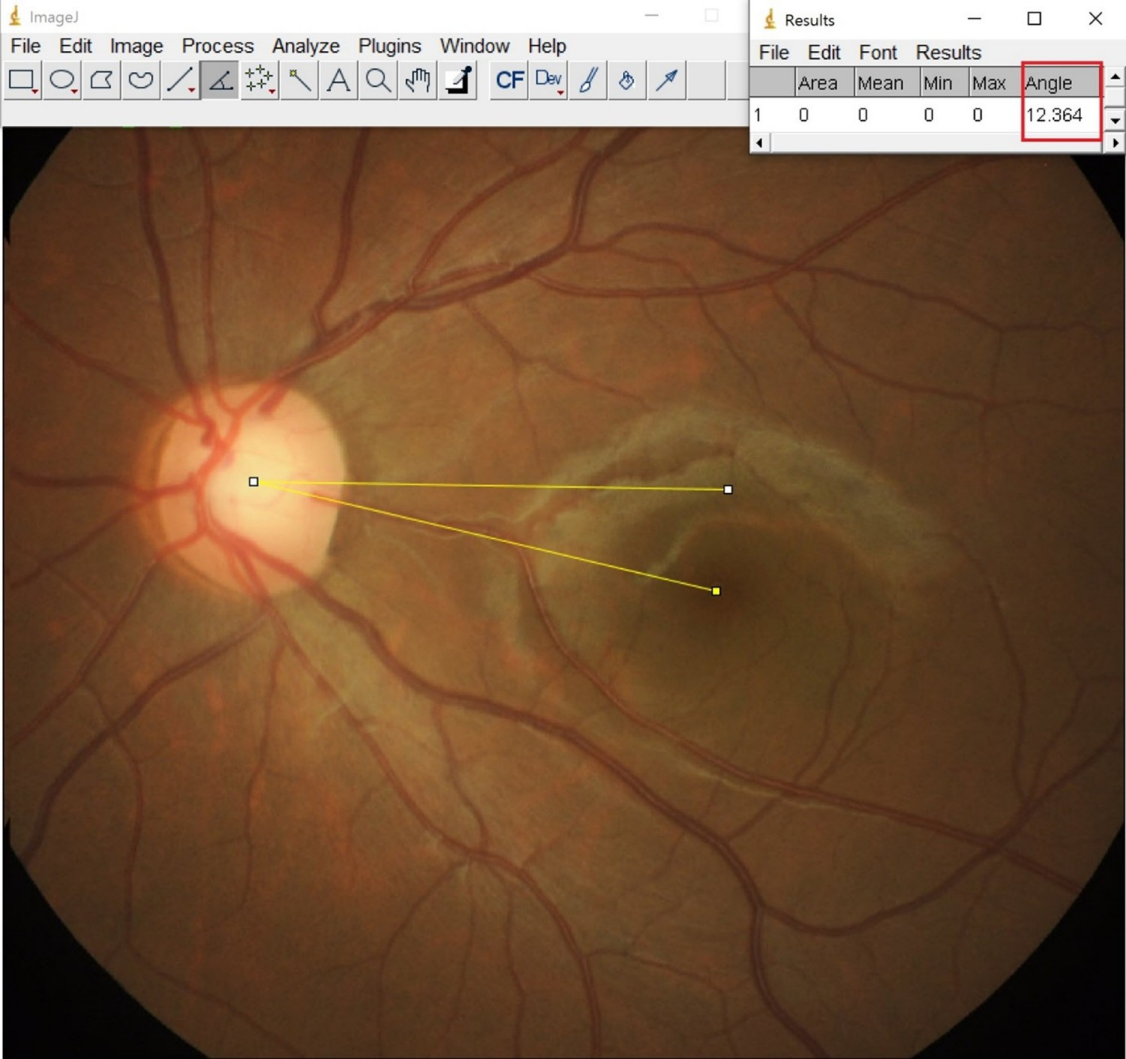
Calculation of the degree of extorsion. Extorsion was evaluated on a fundus photograph in patients with intermittent exotropia and unilateral inferior oblique overaction. The angle of extorsion was measured using Image J software.

The degree of extorsion was calculated using ImageJ software (version 1.8.0, National Institute of Health, Bethesda, MD, USA; Fig 1). In the fundus photographs, the angle between the virtual line connecting the center of the optic disc and the macula and the line passing horizontally through the center of the optic disc was measured manually. In addition, because extorsion may appear in the opposite eye [9], the average torsional angle in both eyes was used to analyze the changes in extorsion. For statistical analysis, extorsion was expressed as a positive value, and intorsion was expressed as a negative value. Enrolled data is in S1 File as supporting information.

Since most patients with IOOA over + 3 had concurrent inferior oblique muscle surgery and horizontal muscle surgery, only patients with + 1 and +2 IOOA were enrolled in this study. The included patients were divided into two groups according to the degree of IOOA: patients with + 1 IOOA were allocated to the +1 IOOA group, while those with + 2 IOOA were allocated to the +2 IOOA group. Changes in the vertical deviation and angle of extorsion before and after surgery in the two groups were compared and analyzed.

### Statistical analysis

Statistical analyses were performed using SPSS software (version 28.0; IBM Corp., Armonk, NY, USA). Baseline characteristics such as sex, age at surgery, type of surgical technique, deviation angle of strabismus, and angle of extorsion between the +1 IOOA and +2 IOOA groups were analyzed using the Mann–Whitney U test or independent t-test. The Wilcoxon signed-rank test was used to analyze the changes in vertical deviation and extorsion after surgery in each group. The change in vertical deviation and extorsion between the two groups was compared using a paired t-test. Statistical significance was set at p < 0.05.

## Results

Forty-one patients with unilateral hypertropia and mild IOOA were included in this study. Seventeen patients were male and 24 were female. The mean age at diagnosis was 8.07 ± 3.44 years (range: 4–14 years) and the mean age at surgery was 9.38 ± 1.04 years (range: 4–16 years). The mean follow-up period was 38.41 ± 3.89 months (range: 1–92 months). The success of the surgery was defined as the horizontal deviation after surgery being within 10 prism diopters (PD), and the surgery success rate was 80% at the final follow up. The mean preoperative horizontal deviation was 25.6 ± 7.28 PD (range: 15–30 PD). The vertical deviation was 2.73 ± 1.33 PD (range: 2–8 PD), and the mean degree of extorsion was 7.53 ± 2.78° (range: 3.40–12.08°). Twenty-four and 17 patients were allocated to the +1 and +2 IOOA groups, respectively. The baseline characteristics of the two groups are described in Table 1. Patients in the two groups did not differ significantly in terms of sex, age at surgery, preoperative stereoacuity, or type of surgical technique. Postoperative fundus imaging was performed at 4.42 ± 5.12 months (range: 1–7 months) in the +1 IOOA group and 4.68 ± 7.84 months (range: 1–8 months) in the +2 IOOA group (p = 0.780). The horizontal deviation between the two groups did not differ before or at any time point after surgery. In terms of vertical deviation, the preoperative values were 2.67 ± 1.52 PD (range: 2–8 PD) in the +1 IOOA group and 2.82 ± 1.13 PD (range: 2–6 PD) in the +2 IOOA group. Although the vertical deviation was numerically larger in the +2 IOOA group than in the +1 IOOA group, the difference was not statistically significant (p = 0.182). The period from operation to postoperative stereoacuity measurement using the FD2 test was 46.75 ± 22.50 months in the +1 IOOA group and 28.60 ± 22.14 months in the +2 IOOA group (p = 0.946). After surgery, stereoacuity showed improvement numerically (from 12.63 ± 1.50 to 10.26 ± 1.17 in + 1 IOOA group, from 19.58 ± 3.87 to 10.83 ± 1.60 in + 2 IOOA group), but without statistical significance, in both groups. Additionally, the values did not differ significantly between the groups (p = 0.318 and p = 0.868, respectively).

**Table 1:** Baseline characteristics of patients.

	+1 IOOA group	+2 IOOA group	p-value
Patients (n)	24	17	
Sex (male:female)	9:15	8:9	0.540*
Age at surgery (year)	9.45±2.12 (4–16) [8, 6.73–8.66]	9.08±7.59 (4–16) [9,7.09–9.97]	0.145^†^
Mean period of fundus photo taken after surgery (months)	4.42±5.12 (1–7) [4, 1.06–5.94]	4.68±7.84 (1–8) [4, 2.24–6.63]	0.780^†^
Surgical techniques			0.513*
BLR Rc	18	14	
ULR Rc	3	1	
R&R	3	2	
Horizontal deviation (PD)			
Preoperative	25.54±5.68 (18–40) [25, 22.97–27.82]	25.65±8.11 (15–35) [25, 19.66–26.00]	0.764^†^
1-day postoperative	-13.13±9.76 (-40 –-2) [-8, -17.66–9.04]	-13.24±4.13 (-25 –-10) [-12, -16.69–10.98]	0.299^†^
1-week postoperative	-5.33±5.29 (-20 to 0) [-6, -2.96 –-7.65]	-5.29±4.04(-14 to 0) [-4, -1.92 –-7.24]	0.820^†^
1-month postoperative	4.92±3.77(-10 to 14) [5, 1.77–6.06]	3.88±4.03 (-10 to 14) [4, -0.82–5.49]	0.247^†^
6-month postoperative	3.83±3.72 (-6 to 10) [4, 1.89–5.41]	3.19±2.97 (-4 to 9) [4, -0.64–4.64]	0.555^†^
Vertical deviation (PD)			
1-day postoperative	2.67±1.52 (2–8) [2, 0.01–0.65]	2.82±1.13(2–6) [2, 0.14–0.64]	0.182^†^
1-week postoperative	0.33±0.76 (0–2) [0, 0.04–0.54]	0.35±0.86 (0–2) [0, 0.08–0.41]	0.936^†^
1-month postoperative	0.25±0.67 (0–2) [0, 0.07–0.41]	0.29±0.77 [0, 0.08–0.41]	0.698^†^
6-month postoperative	0.17±0.56 [0, 0.06–0.31]	0.24±0.56 [0, 0.1–0.27]	0.428^†^
1-day postoperative	0.13±0.44 (0–2) [0, 0.09–0.26]	0.19±0.54 (0–1) [0, 0.1–0.27]	0.671^†^
Frisby Davis distance test (arcsec)			
Preoperative	12.63±1.50 (15, 0–25) [15, 9.48–15.78]	19.58±3.87(15, 5–45) [15, 11.07–28.09]	0.318^‡^
Postoperative	10.26±1.17 (10, 5–20) [10, 7.79–12.74]	10.83±1.60 (10, 5–20) [10, 7.29–14.37]	0.868^‡^
Angle of extorsion (°)			
Preoperative	7.14±2.77 (4.13–12.83) [7.39, 6.27–8.81]	7.94±2.87 (4.56–13.03) [6.49, 5.40–8.90]	0.462^‡^
Postoperative	6.22±2.69 (2.95–11.29) [6.14, 5.28–7.72]	6.71±2.59 (3.07–11.78) [4.72, 4.32–7.41]	0.296^‡^

Values are presented as mean ± standard deviation (Range).

The median and confidence interval are presented in square brackets.

+1 IOOA group: IOOA grade 1; +2 IOOA group: IOOA grade 2.

Patients who underwent reoperation due to recurrence of horizontal strabismus during the follow-up period were excluded.

BLR Rc = bilateral lateral rectus recession; ULR Rc = unilateral lateral rectus recession; R&R = lateral rectus recession and medial rectus resection; IOOA = inferior oblique overaction; PD = prism diopter; ° = degree.*: Chi-square test †: Mann–Whitney test ‡: Independent *t*-test.

Table 2 describes changes in the vertical deviation and angle of extorsion after surgery in the two groups. In both groups, vertical deviation and extorsion were significantly decreased after horizontal muscle surgery (all p < 0.001). In the +1 IOOA group, vertical deviation decreased from 2.67 ± 1.52 PD to 0.17 ± 0.56 PD and extorsion decreased from 7.14 ± 2.77° to 6.22 ± 2.69°. In the +2 IOOA group, vertical deviation decreased from 2.82 ± 1.13 PD to 0.24 ± 0.56 PD and extorsion decreased from 7.94 ± 2.87° to 6.71 ± 2.59°.

**Table 2:** Changes in the vertical deviation and angle of extorsion after surgery.

	Preoperative	Postoperative	p-value
Vertical deviation (PD) (Postoperative 1month)			
+1 IOOA group	2.67±1.52 [2, 2–8]	0.17±0.56 [0, 0–2]	< 0.001^§^
+2 IOOA group	2.82±1.13 [2, 2–6]	0.24±0.56 [0, 0–1]	< 0.001^§^
Angle of extorsion (°)			
+1 IOOA group	7.14±2.77 [7.91, 4.13–12.83]	6.22±2.69 [6.31, 2.95–11.29]	< 0.001^§^
+2 IOOA group	7.94±2.87 [7.99, 4.56–13.03]	6.71±2.59 [6.57, 3.07–11.78]	< 0.001^§^

Values are presented as mean ± standard deviation (Range).

The median and confidence interval are presented in square brackets.

IOOA = inferior oblique overaction; PD = prism diopter; ° = degree.

§: Wilcoxon signed-rank test.

**Fig 2:**
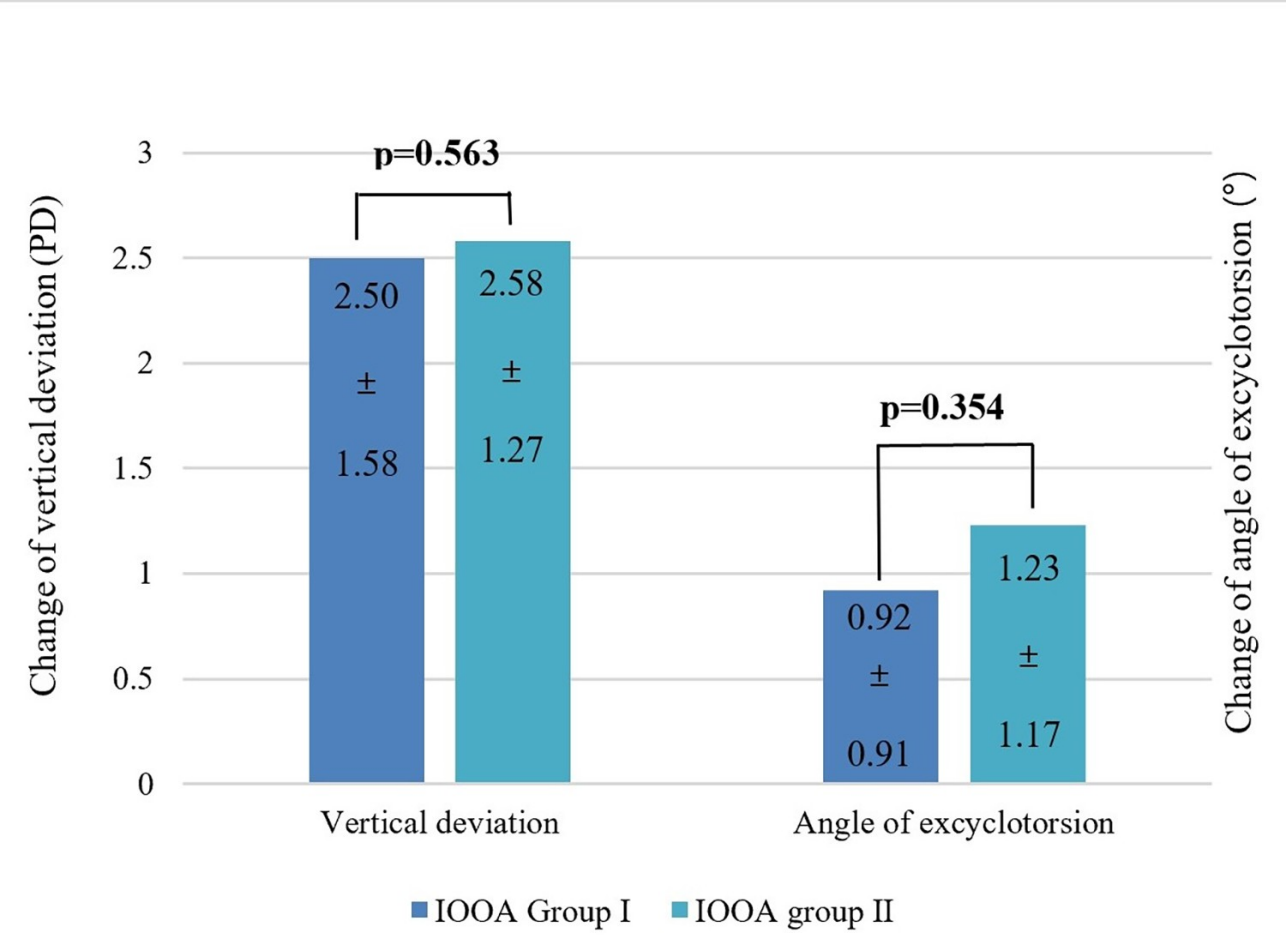
Amount of change in the vertical deviation and angle of extorsion. The amount of change in the vertical deviation and angle of extorsion did not differ statistically significantly between the +1 IOOA group and +2 IOOA group. PD = prism diopter; ° = degree.

Changes in vertical deviation and extorsion were compared (Fig 2). The changes in both vertical deviation and extorsion were larger in the +1 group than in the +2 group; however, the difference was not statistically significant.

## Discussion

This study demonstrated the effects of horizontal muscle surgery on hypertropia and extorsion in patients with exotropia and unilateral IOOA. The results confirmed that hypertropia and extorsion decrease significantly after surgery. In a subanalysis according to the IOOA grade, we found no difference in the amount of change in hypertropia and extorsion between groups after surgery.

Several reports have demonstrated the postoperative course of vertical deviation after horizontal muscle surgery, and the improvement of vertical deviation is debated. Cho and Kim reported that hypertropia decreased from 3.5 PD to 1.2 PD at 1-day postoperatively in their 93 patients; thus, a small amount of hypertropia of up to 14 PD in intermittent exotropia could be ameliorated by horizontal muscle surgery alone [1]. In addition, they stated that surgical correction of horizontal eye alignment can improve not only vertical strabismus but also mild IOOA because vertical and oblique muscle dysfunction might be caused by long-term horizontal eye alignment abnormalities. Similarly, Struck and Daley reported that hypertropia improved after horizontal muscle surgery in 17 patients who underwent horizontal muscle surgery and had superior oblique palsy [10]. In contrast, Cho and Lee suggested that if IOOA is evident in intermittent exotropia coexisting with hypertropia > 4 PD in the primary position, concurrent oblique muscle surgery is needed to correct the hypertropia [11].

Primarily extorsion, secondary elevation, and eyeball abduction are actions of the inferior oblique muscles. IOOA manifests as eye elevation in adduction and is frequently associated with horizontal deviations [12]. It has been reported in 70% of patients with esotropia and 30% of patients with exotropia [13,14]. As the risk of contralateral IOOA after recession of the unilateral IO muscle exists, with an incidence rate of 20–57.1% [14–18], concurrent oblique muscle surgery and horizontal muscle surgery should be carefully considered. Therefore, this study aimed to confirm whether hypertropia and extorsion associated with IOOA and exotropia changed when only horizontal muscle surgery was performed.

As the first result of this study, hypertropia improved significantly in both groups after horizontal muscle surgery, with an overall correction of 2.71 ± 0.24 PD. Our analyzed data corresponded with those of Cho and Kim [1], who reported that hypertropia and mild oblique muscle dysfunction improved only after horizontal eye alignment was corrected. These results also suggest that binocular fusional capacity may be improved by correcting horizontal eye alignment, and consequently, mild hypertropia may be resolved.

As a second result, extorsion also improved significantly in both groups after surgery, and the average correction for the 41 patients overall was 1.14 ± 0.18°. Although the precise mechanisms underlying alterations in ocular torsion after rectus muscle surgery are unclear, two possible mechanisms have been suggested. First, IOOA and the resulting extorsion of the patients included in this study may not have occurred simultaneously with intermittent exotropia but may be an abnormal alignment secondary to long-lasting abduction of the eyeball [19]. Capo et al. [20] provided a mechanical explanation for IOOA in patients with exotropia. They reported that they would expect a patient with exotropia to develop a vertical deviation in extreme gaze into the oblique quadrants, as the abducting eye would reach the mechanical limit, while the adducting eye would still be free to move up or down, resulting in both inferior and superior oblique overaction. In our study, the average age of patients at diagnosis was 8.07 ± 3.44 years, which is older than the average age of exotropia diagnosis (3–6 years) [21,22]. This indicated that intermittent exotropia had been present for a long time in these patients, and as reported by Capo et al., IOOA may have occurred sequentially. Therefore, extorsion due to IOOA may also improve after recovery from exotropia through horizontal muscle surgery.

Secondly, similar to the hypertropia recovery described previously, improvement in binocular fusional capacity after horizontal muscle surgery may induce an improvement in extorsion. In the analysis of stereoacuity, there was a numerically improvement in arcsec in the FD2 test after surgery in each group, which implies an improvement of fusional capacity. Although statistical significance was not satisfied, it is considered a limitation of this study due to the small number of samples, and a further study with a larger number of patients would be meaningful.

Changes in the vertical deviation and extorsion did not differ significantly between the two groups. This indicates the absence of a sensitive effect on torsion in mild IOOA ≤ grade 2 [23]. An additional study on the effects of horizontal muscle surgery on ocular torsion changes in severe IOOA of ≥ grade 3 will be helpful.

This study had some limitations. The sample size was small, and the time at which fundus photography was performed to calculate extorsion after surgery was not unified (from 1 to 8 months postoperatively). Although the groups did not differ significantly in terms of the time at which the fundus photo was taken, timing variations may have influenced the study outcomes. As another limitation, prism control score was only performed on some patients and data for all patients was not obtained. Additionally, sub-analysis according to primary and secondary IOOA could not be performed due to the small sample size. Further studies using a large number of patients are warranted.

Nevertheless, this study is meaningful because it reports that ocular torsion can change even after horizontal muscle surgery alone in patients with intermittent exotropia coexisting with unilateral IOOA. In conclusion, in 41 patients with exotropia with an average of 25.59 ± 7.28 PD and IOOA ≤ grade 2, hypertropia decreased significantly by 2.71 ± 0.24 PD; ocular torsion also decreased by 1.14 ± 0.18° after surgery.

## Supporting information

S1 File (XLSX)
